# S-Nitrosothiols modulate G protein-coupled receptor signaling in a reversible and highly receptor-specific manner

**DOI:** 10.1186/1471-2121-6-21

**Published:** 2005-04-25

**Authors:** Tarja Kokkola, Juha R Savinainen, Kati S Mönkkönen, Montse Durán Retamal, Jarmo T Laitinen

**Affiliations:** 1 Department of Physiology, University of Kuopio, POB 1627, FIN-70211, Kuopio, Finland; 2 Department of Pharmaceutical Chemistry, University of Kuopio, POB 1627, FIN-70211 Kuopio, Finland

## Abstract

**Background:**

Recent studies indicate that the G protein-coupled receptor (GPCR) signaling machinery can serve as a direct target of reactive oxygen species, including nitric oxide (NO) and S-nitrosothiols (RSNOs). To gain a broader view into the way that receptor-dependent G protein activation – an early step in signal transduction – might be affected by RSNOs, we have studied several receptors coupling to the G_i _family of G proteins in their native cellular environment using the powerful functional approach of [^35^S]GTPγS autoradiography with brain cryostat sections in combination with classical G protein activation assays.

**Results:**

We demonstrate that RSNOs, like S-nitrosoglutathione (GSNO) and S-nitrosocysteine (CysNO), can modulate GPCR signaling via reversible, thiol-sensitive mechanisms probably involving S-nitrosylation. RSNOs are capable of very targeted regulation, as they potentiate the signaling of some receptors (exemplified by the M2/M4 muscarinic cholinergic receptors), inhibit others (P2Y_12 _purinergic, LPA_1_lysophosphatidic acid, and cannabinoid CB_1 _receptors), but may only marginally affect signaling of others, such as adenosine A_1_, μ-opioid, and opiate related receptors. Amplification of M2/M4 muscarinic responses is explained by an accelerated rate of guanine nucleotide exchange, as well as an increased number of high-affinity [^35^S]GTPγS binding sites available for the agonist-activated receptor. GSNO amplified human M4 receptor signaling also under heterologous expression in CHO cells, but the effect diminished with increasing constitutive receptor activity. RSNOs markedly inhibited P2Y_12 _receptor signaling in native tissues (rat brain and human platelets), but failed to affect human P2Y_12 _receptor signaling under heterologous expression in CHO cells, indicating that the native cellular signaling partners, rather than the P2Y_12 _receptor protein, act as a molecular target for this action.

**Conclusion:**

These in vitro studies show for the first time in a broader general context that RSNOs are capable of modulating GPCR signaling in a reversible and highly receptor-specific manner. Given that the enzymatic machinery responsible for endogenous NO production is located in close proximity with the GPCR signaling complex, especially with that for several receptors whose signaling is shown here to be modulated by exogenous RSNOs, our data suggest that GPCR signaling in vivo is likely to be subject to substantial, and highly receptor-specific modulation by NO-derived RSNOs.

## Background

G protein-coupled receptors (GPCRs) represent the largest group of integral membrane proteins involved in signal transduction and are the most important targets of clinically marketed drugs [1-3]. The known GPCRs mediate messages from ligands as diverse as neurotransmitters, lipid mediators, hormones, and sensory stimuli [4]. The classical scheme of GPCR signaling implies that agonist-induced conformational changes in receptor molecule will result in activation of cognate G proteins and subsequently in the regulation of downstream effectors, second messengers, and the activation of protein kinases, for example [4]. However, recent work has indicated that GPCR signaling is subject to complex, cell-type specific regulation, involving a plethora of kinases, as well as newly-identified signaling partners, such as regulators of G protein signaling (RGS) [5], and activators of G protein signaling (AGS) [6].

Nitric oxide (NO) is a unique gaseous messenger generated in vivo by three isoforms of NO synthases (NOS). The established mode of NO signaling is through the activation of the hemoprotein, soluble guanylyl cyclase, resulting in increased production of the second messenger cGMP. However, accumulating evidence points towards cGMP-independent mechanisms by which NO can react with proteins, forming covalent post-translational modifications [7]. S-nitrosothiols (RSNOs) are biological metabolites of NO, that may prolong and spatially extend the in vivo actions of locally produced NO [8]. NO and RSNOs can reversibly react with free SH-groups of target cysteine (Cys) residues, including those in proteins, leading to S-nitrosylation and/or S-thiolation (disulfide linkage of low-molecular weight thiols to proteins) [8-17]. A broad functional spectrum of potential S-nitrosylation target proteins is currently recognized. A growing list of targets include ion channels, transporters, transcription factors, signaling proteins, metabolic enzymes, as well as respiratory proteins [7,14,18-20]. Although individual components of the GPCR signaling machinery are implicated as potential targets of reactive oxygen species (ROS), including NO [21-35], a broader view on how NO, and RSNOs in particular, might modulate GPCR signaling, has not been established.

To begin to address these issues, we have studied how exogenous RSNOs affect receptor-mediated G protein activity – a very proximal step of GPCR signal transduction – by studying the signaling of several receptors that couple to the G_i _family of heterotrimeric G proteins. This family consists of both pertussis toxin sensitive (Gα_i1-3_, Gα_o_, transducin, gustducin) and insensitive (Gα_z_) members. We applied the powerful functional approach of [^35^S]GTPγS autoradiography in brain cryostat sections, as this technique allows selective detection of receptor-dependent G protein activity simultaneously in multiple brain regions with minimal disturbance of the GPCR microenvironment [36]. We anticipated that accessibility of target thiols would be minimally disturbed in cryostat sections. Moreover, it is increasingly recognized that specialized plasma membrane microdomains (variously described as detergent-resistant fractions, low-density fractions, lipid rafts, or caveolae) act as unique signaling platforms with specific enrichment of GPCRs, their cognate G proteins, as well as effectors [37-40]. Such an enrichment is thought to be well-preserved in cryostat sections, but might be compromised to a variable extent [38], or even lost in bulk membrane preparations obtained using traditional protocols. To complement the autoradiography approach, we used membrane and lysate [^35^S]GTPγS binding assays to more systematically study the effects of RSNOs on a panel of G_i_-coupled receptors in native tissues. Signaling of selected receptors was further studied after their heterologous expression in Chinese hamster ovary (CHO) cells. Our studies reveal highly receptor-specific modulation of GPCR signaling by RSNOs, as signaling of some receptors can be amplified, or inhibited, whereas for others, the activity is only marginally affected by comparable treatments. The GPCR itself and/or its native signaling partners seem to act as potential targets of RSNO action, and therefore their modulation may be diminished, or even totally masked under heterologous expression.

## Results

### Exogenous RSNOs modulate GPCR signaling via mechanisms likely involving S-nitrosylation

**Figure 1:**
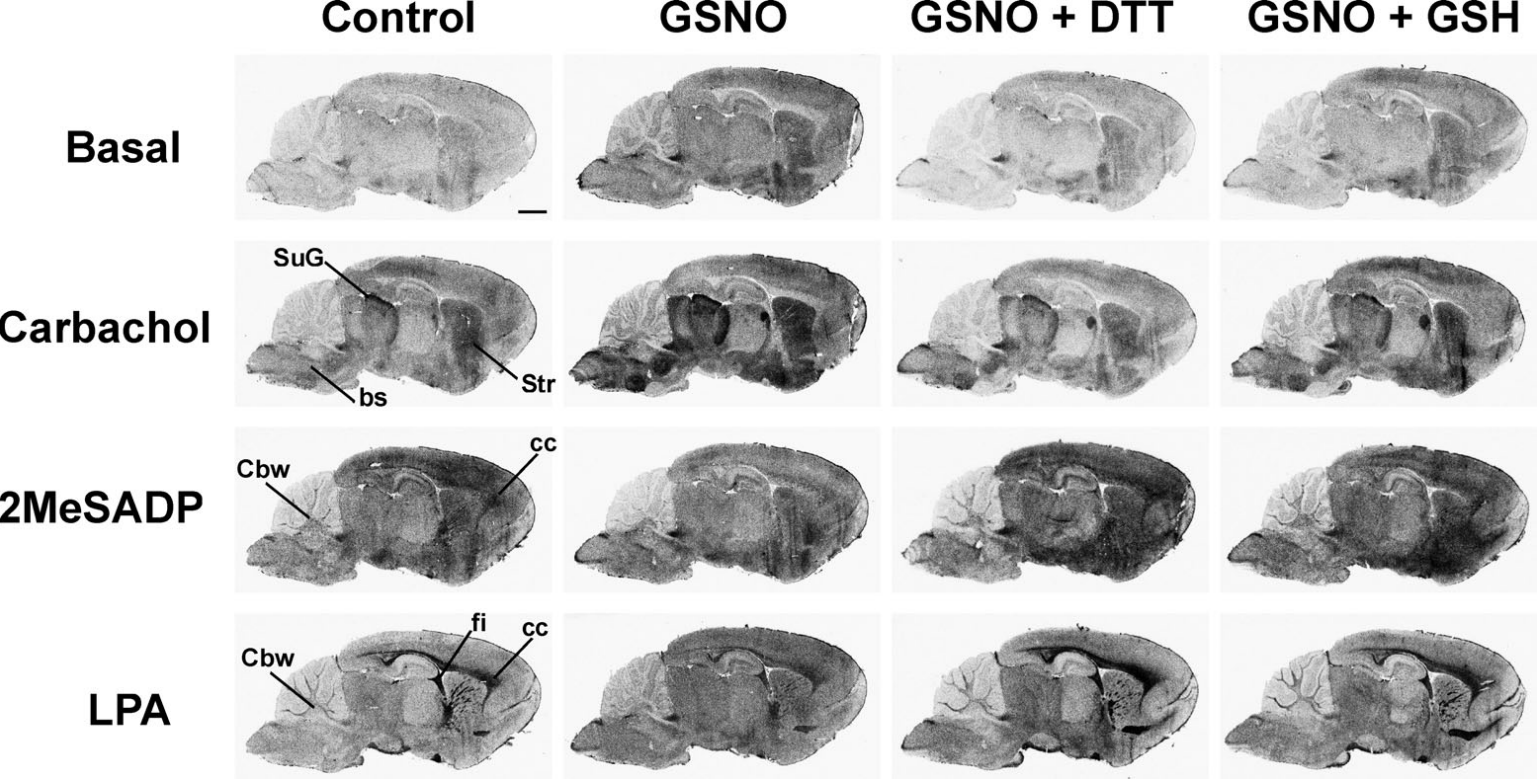
**S-nitrosoglutathione (GSNO) reversibly modulates basal and receptor-dependent G protein activity in rat brain cryostat sections**. [^35^S]GTPγS autoradiography of sagittal brain sections was conducted using a 3-step protocol with DPCPX (10^-6 ^M) present throughout steps 2 and 3, as detailed in the *Methods *section. Where indicated, GSNO (0.5 mM) was present for 60 min during the GDP loading (step 2). When used, DTT (1 mM) or GSH (1 mM) were present during the [^35^S]GTPγS labeling (step 3). The muscarinic agonist, carbachol (CCh, 10^-4 ^M), the P2Y receptor agonist 2-methylthio-ADP (2MeSADP, 10^-5 ^M) or lysophosphatidic acid (LPA, 5 × 10^-5 ^M in 0.1% fatty acid free BSA) were present in step 3. In the control panel (left), the anatomical loci where receptor agonists typically activate G proteins are indicated. Note GSNO-dependent overall increase in basal G protein activity, as well as robust amplification of CCh-stimulated G protein activity in several gray matter regions visible at this sagittal plane, most notably the brain stem (bs) nuclei, the striatum (Str), and the superficial gray layer of the superior colliculus (SuG). Note also clear attenuation of 2MeSADP-stimulated responses in all brain regions, and blunting of LPA-stimulated responses, especially in the white matter areas, including the corpus callosum (cc), the fimbria of the hippocampus (fi) and the cerebellar white matter (Cbw). Scale bar = 2 mm. For quantitative data on selected brain regions, see Supplementary Figs. 1 and 2 in additional file 1.

We used the functional approach of [^35^S]GTPγS autoradiography, as this technique allows selective detection of receptor-stimulated G_i _protein activity simultaneously in multiple brain structures with minimal disturbance of the GPCR microenvironment [36]. We focused the initial experiments on three G_i_-linked receptors, namely M2/M4 AChRs, the P2Y_12 _purinoceptor, and the LPA_1 _receptor, as G protein activity upon stimulation of these receptors has been previously characterized using the autoradiography approach and each receptor shows a unique anatomical distribution pattern in the developing rat brain [36,41-43]. As depicted in Figure 1, pretreatment of brain sections with freshly prepared GSNO (0.5 mM) had distinct effects on basal and receptor-stimulated [^35^S]GTPγS binding responses. In GSNO-treated sections, basal G protein activity was increased throughout the gray matter areas and this effect was fully reversed in the presence of excess thiol, either in the form of dithiotreitol (DTT) or reduced glutathione (GSH). The autoradiography images were quantified for selected brain regions and these results are shown in Supplementary Figures 1 and 2 [see additional file 1]. Consistent with the anatomical distribution of atropine-sensitive and M2/M4 AChR-dependent G protein activity [36,44,45], the cholinergic agonist carbachol (CCh) stimulated [^35^S]GTPγS binding to multiple gray matter regions, including the striatum (Str), the thalamic structures, with the most intense responses in the superficial gray layer of the superior colliculus (SuG), as well as various brainstem (bs) nuclei. In all visible regions, CCh-stimulated G protein activity was robustly amplified by GSNO. This was particularly evident in the above-mentioned M2/M4 receptor-enriched anatomical loci. It is noteworthy that the GSNO effects were fully reversed in all regions in the presence of DTT or GSH (Figure 1, Supplementary Figure 1 [see additional file 1]). As further illustrated in Figure 1, the P2Y receptor agonist 2-methylthio-ADP (2MeSADP) activated G proteins both in gray and white matter regions, producing a heterogeneous activity pattern with characteristic "hot spot" appearance, as described earlier [41,42]. Previous studies have established that the 2MeSADP-stimulated G protein activity in rat brain sections is mediated by a P2Y receptor subtype that pharmacologically corresponds to P2Y_12 _[41,42]. In contrast to the robust amplification of M2/M4 receptor-dependent G protein activity, GSNO clearly inhibited P2Y_12 _receptor signaling (Figure 1, Supplementary Figures 1 and 2 [see additional file 1]). This inhibition was evident throughout the effective agonist concentration range (10^-7 ^-10^-4 ^M 2MeSADP), and responses to the endogenous agonist ADP (5 × 10^-5 ^- 10^-3 ^M) were similarly blunted (Supplementary Figure 2 [see additional file 1]). The GSNO-effect was fully reversible upon addition of DTT or GSH (Figure 1, Supplementary Figure 1 [see additional file 1]). In the developing rat brain, LPA-stimulated G_i _protein activity is largely restricted to the myelinating white matter tracts [36,41,43] (Figure 1), closely reflecting the anatomical distribution of LPA_1 _receptor subtype [Ref. 36, and references therein]. Similar to the P2Y_12 _responses, LPA_1 _receptor responses were suppressed in GSNO-treated sections throughout the white matter tracts. Also this effect was fully reversed in the presence of thiols (Figure 1, Supplementary Figure 1 [see additional file 1]).

**Figure 2:**
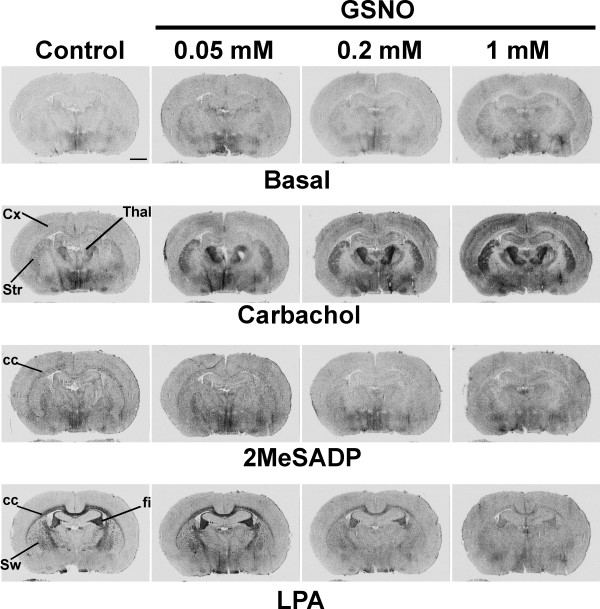
**GSNO modulates GPCR signaling in a dose-dependent manner**. [^35^S]GTPγS autoradiography was conducted using a 3-step protocol with DPCPX (10^-6 ^M) present throughout steps 2 and 3, as detailed in the *Methods *section. GSNO was present at the indicated concentrations for 60 min during the GDP loading (step 2). Carbachol (CCh, 10^-4 ^M), 2MeSADP (10^-6 ^M) or LPA (5 × 10^-5 ^M in 0.1% fatty acid free BSA) were present in step 3. Note dose-dependent amplification of CCh-stimulated G protein activity, most evident at this coronal plane in the cerebral cortex (Cx), the striatum (Str), and the thalamus (Thal). Note also dose-related attenuation of 2MeSADP- and LPA-stimulated responses, especially in the white matter regions, including the corpus callosum (cc), the fimbria of the hippocampus (fi), and the striatal white matter (Sw). Scale bar = 2 mm. For quantitative data on selected brain regions, see Supplementary Figs. 1 and 2 in additional file 1.

GSNO is present in significant amounts (~15 pmol/mg protein) in the brain tissue and it is thought to act as a physiological carrier of NO for S-nitrosylation reactions [46,47]. As shown in Figure 2 and Supplementary Figure 1 [see additional file 1], GSNO modulated GPCR responses in a dose-dependent manner, being effective at the submillimolar concentration range. However, the threshold and maximal concentrations needed for the modulation of distinct receptors slightly varied. Potentiation of M2/M4 receptor signaling was evident already with 0.05 mM GSNO, but statistically significant responses were obtained using 0.2 – 1 mM GSNO (Supplementary Figure 1 [see additional file 1]). P2Y_12 _receptor responses were inhibited with 0.2 mM GSNO and higher concentrations (Figure 2, Supplementary Figure 1 [see additional file 1]). LPA_1 _receptor signaling was only marginally affected with 0.05 mM GSNO, but was severely blunted with 0.2 mM GSNO and higher concentrations (Figure 2, Supplementary Figure 1 [see additional file 1]). Collectively, these experiments demonstrate that GSNO, a physiologically relevant RSNO, can modulate GPCR signaling in discrete brain regions in a receptor-specific and fully reversible manner.

**Figure 3:**
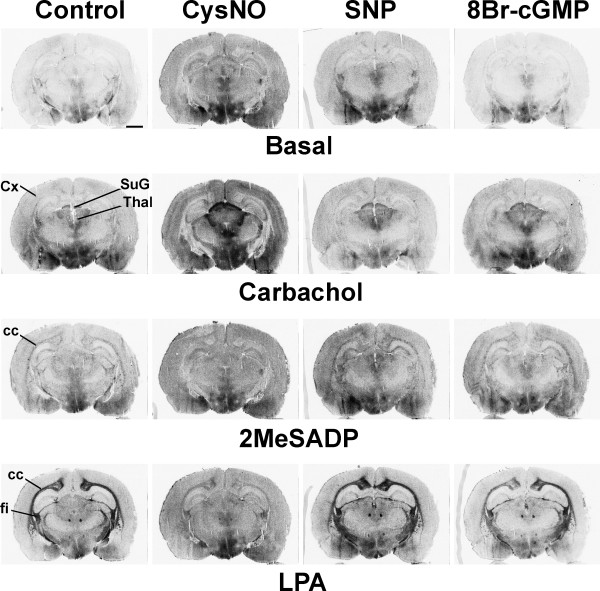
**S-nitrosocysteine (CysNO) mimics the effects of GSNO in modulating GPCR signaling, whereas sodium nitroprusside (SNP) and 8-bromo cyclic GMP (8Br-cGMP) do not**. [^35^S]GTPγS autoradiography was conducted using a 3-step protocol with DPCPX (10^-6 ^M) present throughout steps 2 and 3, as detailed in the *Methods *section. Where indicated, CysNO (1 mM), SNP (0.5 mM), or 8Br-cGMP (0.25 mM) were present for 60 min during the GDP loading (step 2). Carbachol (10^-4 ^M), 2MeSADP (10^-6 ^M), or LPA (5 × 10^-5 ^M in 0.1% fatty acid free BSA) were present in step 3. Note amplification of CCh-stimulated G protein activity by CySNO, most evident at this coronal plane in the cerebral cortex (Cx), the thalamus (Thal), including the superficial gray layer of the superior colliculus (SuG). Note also CysNO-dependent attenuation of 2MeSADP- and LPA-stimulated responses, most evident in the corpus callosum (cc) and the fimbria of the hippocampus (fi). Scale bar = 2 mm. For quantitative data on selected brain regions, see Supplementary Table 1 in additional file 1.

Various RSNOs, including S-nitrosocysteine (CysNO) (Figure 3, Supplementary Table 1 [see additional file 1]), S-nitrosocysteamine, S-nitroso-L-cysteinylglycine (CysNO-Gly), L-γ-glutamyl-S-nitrosocysteine (Glu-CysNO), and S-nitroso-N-acetyl-D,L-penicillamine (SNAP) – a compound with a sterically hindered SNO group – mimicked the effects of GSNO on the three studied receptors (Figure 3, Supplementary Figure 2 [see additional file 1], and data not shown). When equimolar concentrations (0.5 mM) of GSNO and SNAP were compared, SNAP equally well suppressed P2Y_12 _receptor responses, whereas GSNO more efficiently modulated M2/M4 and LPA_1 _receptor responses (Supplementary Figure 3 [see additional file 1]). The modulatory effect of CysNO on the three receptors was reversed by addition of DTT or cysteine (Supplementary Figure 4 [see additional file 1]). However, when "aged" CysNO was used (the stock solution was left to stand for 2 days in ambient light, oxygen and temperature), receptor-stimulated G protein activity was almost completely abolished (Supplementary Figure 5 [see additional file 1]).

**Table 1:** Effects of GSNO treatment on agonist dose-response parameters in [^35^S]GTPγS binding assays of various G_i_-coupled receptors in their native cellular environment. Membranes or lysates were preincubated in control conditions or in the presence of 0.5 mM GSNO for 30 min. Values are mean ± SE from three to four independent experiments performed in duplicate. E_max _is expressed in percentage over basal with nonspecific binding subtracted.

	Control		GSNO	
		
Receptor (agonist)	log(EC_50_)	E_max _(%)	log(EC_50_)	E_max _(%)
***Rat forebrain membranes***				
M2/M4 mAChRs (CCh)	-4.94 ± 0.05	176 ± 2	-5.55 ± 0.11**	207 ± 4**
LPA_1 _(LPA)	-6.84 ± 0.26	140 ± 7	-6.53 ± 0.40	116 ± 3*
Cannabinoid CB_1 _(CP55940)	-7.84 ± 0.07	223 ± 3	-7.69 ± 0.08	183 ± 2***
Adenosine A_1 _(2ClAdo)	-6.90 ± 0.04	256 ± 3	-6.87 ± 0.12	234 ± 7*
μ-opiate (DAMGO)	-6.85 ± 0.09	167 ± 3	-7.31 ± 0.19	164 ± 5
ORL1 (Nociceptin)	-8.66 ± 0.12	173 ± 3	-8.78 ± 0.10	161 ± 2*
				
***CHO cell lysates***				
LPA (LPA)	-7.37 ± 0.11	202 ± 4	-7.05 ± 0.12	176 ± 4**
				
***Human platelet membranes***				
P2Y_12 _(MeSADP)	-8.19 ± 0.03	279 ± 2	-8.30 ± 0.08	195 ± 3***
α_2A_-adrenoceptor (NA)	-5.33 ± 0.08	161 ± 2	-5.49 ± 0.09	182 ± 3**

* Statistically different from control (P < 0.05)

** Statistically different from control (P < 0.01)

*** Statistically different from control (P < 0.001)

**Figure 4:**
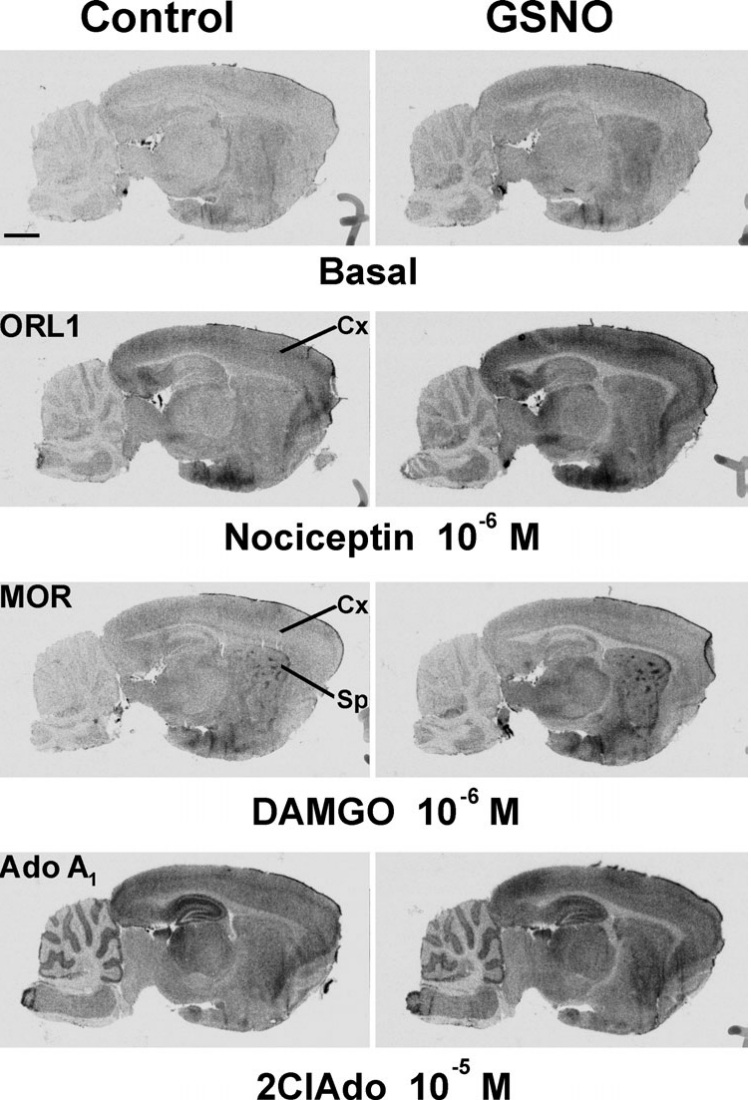
**GSNO only marginally affect opiate related receptor (ORL1), μ opioid receptor (MOR) and adenosine A_1 _(Ado A_1_) receptors signaling in brain sections**. [^35^S]GTPγS autoradiography was conducted using a 3-step protocol with adenosine deaminase (ADA, 1 U/ml) present throughout steps 2 and 3, as detailed in the *Methods *section. Where indicated, GSNO (1 mM) was present for 60 min during the GDP loading (step 2). Protease inhibitor cocktail was included in step 2 for brain sections used for testing ORL1 and MOR responses. Receptor agonists nociceptin (ORL1), DAMGO (MOR), and 2-chloroadenosine (2ClAdo) (adenosine A_1 _receptor) were present at submaximal concentrations during step 3. Note wide distribution of nociceptin-responsive brain regions, including the cerebral cortex (Cx). Note also robust response to DAMGO in the MOR-enriched striatal patches (Sp), as well as the relatively GSNO-resistant, and widely distributed adenosine A_1 _receptor-dependent signal throughout the sagittal plane. Scale bar = 2 mm. For quantitative data on selected brain regions, see Supplementary Table 2 in additional file 1.

**Figure 5:**
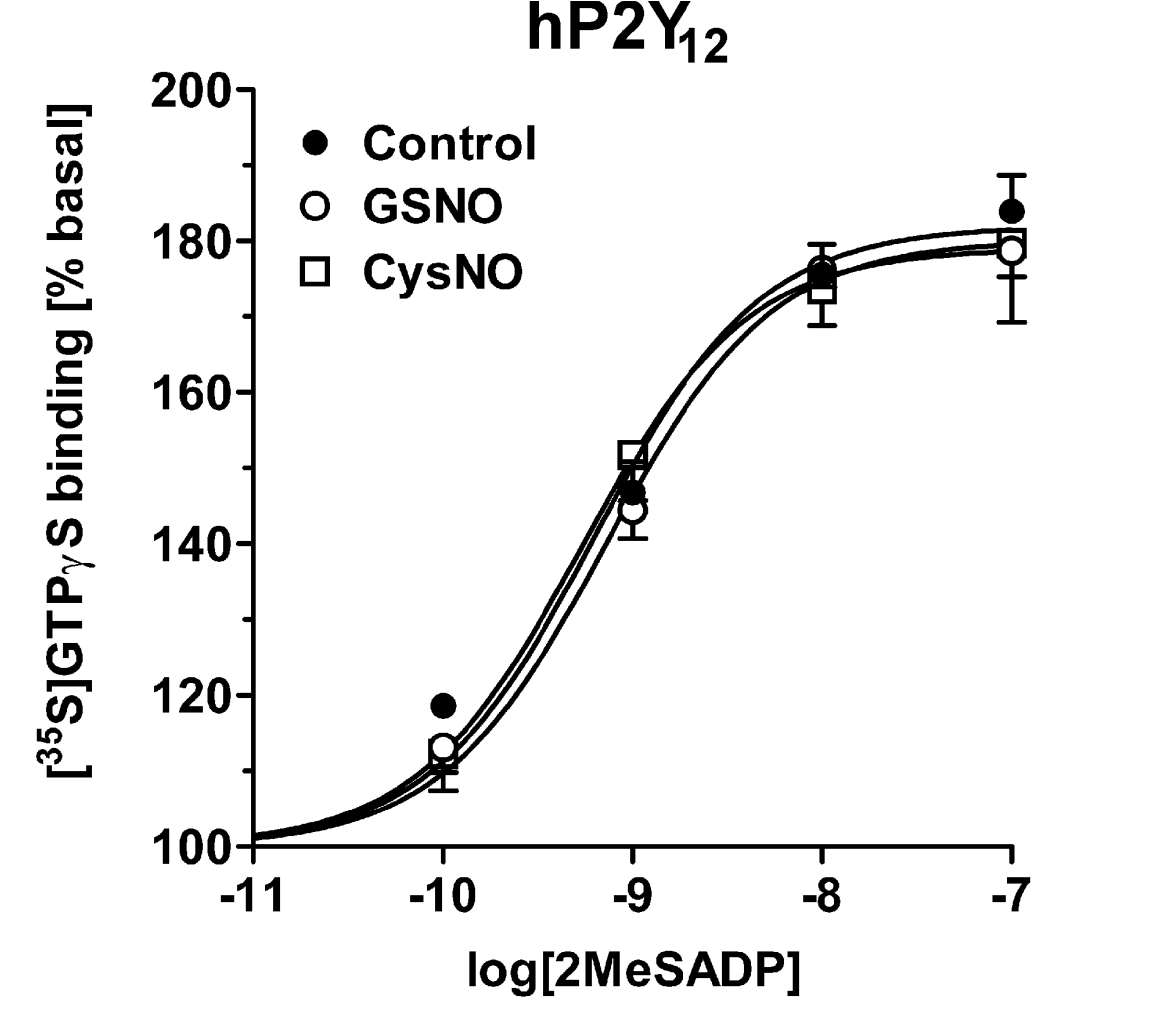
**The P2Y_12 _receptor is not a direct target of RSNO action**. Human P2Y_12 _receptor was stably expressed in CHO cells and agonist-stimulated G protein activity was determined in control conditions and in membranes pretreated for 30 min with 0.5 mM GSNO or CysNO, as detailed in the *Methods *section. There were no statistical differences in the agonist potency [log (EC_50_): control -9.20 ± 0.09; GSNO -9.14 ± 0.07; CysNO -9.27 ± 0.14) or efficacy [E_max _(% basal): control 182 ± 3 %; GSNO 180 ± 2; CysNO 179 ± 5%]. Values are mean ± SE from three independent experiments performed in duplicate.

Previous studies have suggested that RSNOs can act as NO^+^, NO^.^, and NO^- ^donors under physiological conditions [9]. The RSNO effects on GPCR responses were not mimicked by the NO donor sodium nitroprusside (SNP), when tested at similar (0.5 mM) concentrations (Figure 3, Supplementary Table 1 [see additional file 1]). Furthermore, the NO^+^-releasing NO donor, nitrosodium tetrafluoroborate (NOBF_4_, 0.5 mM) (data not shown), hydrogen peroxide (1 mM H_2_O_2 _+ 0.2 mM FeSO_4_) (data not shown), or the cyclic GMP analog 8-bromo-cyclic GMP (0.25 mM) (Figure 3, Supplementary Table 1 [see additional file 1]) were largely ineffective. For selected brain regions, quantitative autoradiography data on CysNO, SNP and 8-bromo-cyclic GMP are presented in the Supplementary Table 1 [see additional file 1].

The above results demonstrate that the effects of NO-related species was shared by -and restricted to – different classes of RSNO compounds, suggesting that S-nitrosylation rather than other types of NO reactions, or cGMP-dependent mechanisms, were involved. According to the S-nitrosylation scheme, treatment with exogenous RSNOs should result in transnitrosylation of potential protein thiols (R-SNO + Protein-SH ↔ R-SH + Protein-SNO). To demonstrate the presence of SNO moieties in GSNO-treated brain section, we used the indirect approach where heterolytic cleavage of S-NO bond with HgCl_2 _generates nitrite which can be measured by a colorimetric method. To this end, brain sections were treated with GSNO (0.5 mM), and after thorough washes, the sections were incubated further in the absence or presence of HgCl_2 _(10^-4 ^M). These experiments (shown in Supplementary Figure 6 [see additional file 1]) revealed that HgCl_2_-catalyzed nitrite formation was significantly higher in GSNO treated sections than that in control conditions (2.46 ± 0.13 vs. 0.61 ± 0.14 nmol NO_2_^- ^per four coronal brain sections, mean ± SE, n = 3, P < 0.001). Collectively these experiments suggest that the RSNO-evoked and receptor-specific modulation of GPCR signaling in brain sections most likely involves S-nitrosylation mechanisms.

**Figure 6:**
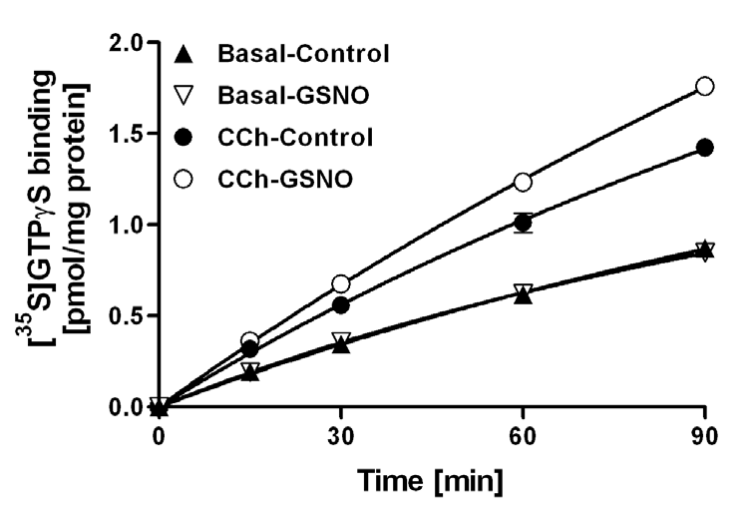
**GSNO accelerates the rate of M2/M4 AChR-stimulated [^35^S]GTPγS binding to rat forebrain membranes but has no effect on basal guanine nucleotide exchange rate**. Membranes were preincubated for 30 min in control conditions or in the presence of 0.5 mM GSNO, and time-response for basal and CCh-stimulated (10^-4 ^M] [^35^S]GTPγS binding was determined, as detailed in the *Methods *section. Values represent specific binding (mean ± SD of duplicate determinations) from one representative experiment that was replicated three times with similar outcome.

### RSNOs modulate GPCR signaling in native tissues in a highly receptor-specific manner

**Figure 7:**
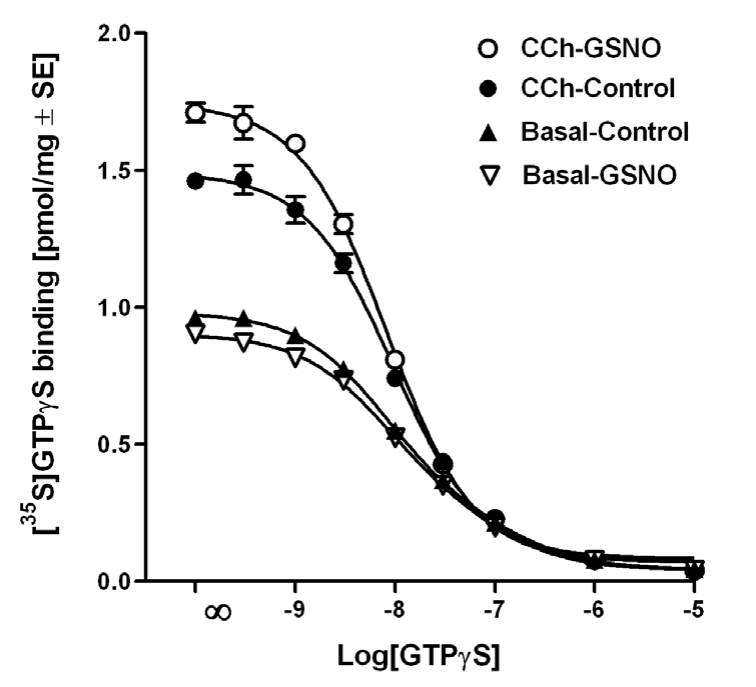
**GSNO increases the number of M2/M4 AChR interacting high-affinity [^35^S]GTPγS binding sites in rat forebrain membranes**. Membranes were preincubated for 30 min in control conditions or in the presence of 0.5 mM GSNO, and incubated thereafter for 90 min with 0.15 nM [^35^S]GTPγS, 10^-5 ^M GDP and indicated concentrations of unlabeled GTPγS in the presence and absence of CCh (10^-4 ^M), as detailed in the *Methods *section. Statistical comparison of one- versus two-site competition curves (nonlinear regression) indicated that the one-site model best described GTPγS displacement in agonist-stimulated conditions. Note that GSNO significantly increased the number of high-affinity [^35^S]GTPγS binding sites in CCh-treated membranes (CCh-control 1.49 ± 0.02; CCh-GSNO 1.75 ± 0.02 pmol/mg protein, P < 0.001, unpaired T-test). There were no statistical differences in the potency for GTPγS in displacing radioligand in any condition [log (EC_50_): basal-control -8.10 ± 0.07; basal-GSNO -8.11 ± 0.12; CCh-control -8.06 ± 0.06; CCh-GSNO -8.10 ± 0.04). Values represent specific binding (mean ± SE) from three independent experiments performed in duplicate.

Although [^35^S]GTPγS autoradiography offers the advantage of monitoring G protein activity simultaneously in multiple brain regions with minimal disturbance of the GPCR microenvironment, generating quantitative data from the autoradiography images is relatively tedious. As a complementary approach, we tested the effect of RSNOs on agonist potency and efficacy for several additional G_i_-coupled receptors using classical membrane and lysate [^35^S]GTPγS binding assays. The results of these experiments are summarized in Table 1. There was one major difference from the situation with brain sections; in the rat forebrain membrane preparations GSNO did not significantly affect basal G protein activity (102 ± 2 % control, mean ± SE, n = 26, see also Figures 6 and 7). However, GSNO modulation of receptor-mediated responses was found to be highly receptor-specific. In line with the autoradiography data, M2/M4 receptor signaling was markedly amplified (both the potency and the efficacy of agonist increased) whereas LPA-evoked signaling efficacy (but not agonist potency) was significantly decreased in the rat forebrain membrane preparations. Moreover, the efficacy of cannabinoid CB_1 _receptor signaling was significantly inhibited with no concomitant change in agonist potency (Table 1). [^35^S]GTPγS autoradiography studies further indicated that CB_1 _receptor signaling was similarly inhibited in various CB_1 _receptor-enriched brain regions, including the cerebral cortex, the hippocampus and the globus pallidus (Supplementary Figure 7 [see additional file 1]). On the other hand, signaling via other widely distributed receptors, such as adenosine A_1_, μ-opioid (MOR) and opiate-related receptor (ORL1), was only marginally (A_1 _and ORL1), or not detectably (MOR), altered in bulk membrane preparations (Table 1) or in brain cryostat sections (Figure 4). For the three receptors, quantitative autoradiography data on selected brain regions are presented in Supplementary Table 2 [see additional file 1].

**Table 2:** Effects of GSNO treatment on agonist (CCh) dose-response parameters in [^35^S]GTPγS binding assays of hM4 cell line membranes. Membranes were preincubated in control conditions or in the presence of 0.5 mM GSNO for 30 min. Values are mean ± SE from three to four independent experiments performed in duplicate. E_max _is expressed in percentage over basal with nonspecific binding subtracted.

	Control		GSNO	
		
CHO cell line	log(EC_50_)	E_max _(%)	log(EC_50_)	E_max _(%)
hM4-WT-A1	-4.87 ± 0.14	586 ± 31	-4.94 ± 0.09	674 ± 23
hM4-WT-E5	-4.87 ± 0.05	213 ± 3	-4.98 ± 0.13	276 ± 10**
hM4-WT-C2	-5.21 ± 0.20	134 ± 3	-5.27 ± 0.20	154 ± 8*
hM4-C133S-H2	-5.13 ± 0.10	198 ± 4	-5.12 ± 0.11	235 ± 6**

* Statistically different from control (P < 0.05)

** Statistically different from control (P < 0.01)

CHO cells express endogenous G_i_-coupled LPA receptors [48-50], and similarly to the situation in brain membranes and cryostat sections, GSNO significantly inhibited LPA signaling in CHO cell lysates without affecting the agonist potency (Table 1). Platelets offer another readily accessible model to study P2Y_12_-G_i _(specifically Gα_i2_) signaling in native cellular environments [42,51,52]. In human platelet membranes, GSNO inhibited basal G protein activity by 22 ± 4 % (mean ± SE, n = 3). This effect was statistically significant. Similar to the situation with brain P2Y_12 _receptor signaling, GSNO inhibited P2Y_12 _receptor-dependent G protein activity in human platelet membranes, but had no effect on the agonist potency (Table 1). To examine whether the receptor protein serves as a direct target of this action, human P2Y_12 _receptor was stably transfected into CHO cells. Several cell lines responding to 2MeSADP in [^35^S]GTPγS binding assays were established (our unpublished observations). However, neither GSNO nor CysNO affected the 2MeSADP dose-response curves of any of the hP2Y_12_-expressing cell lines (Figure 5 and data not shown). These data rule out the P2Y_12 _receptor protein as a direct target of the RSNO action.

The inhibitory effect of GSNO on basal and P2Y_12 _receptor-dependent G protein activity was not due to a nonspecific action on platelet membranes, since the signaling of another G_i_-linked platelet receptor, the α_2A_-adrenoceptor, was significantly amplified in GSNO-treated membranes (Table 1). This effect was not restricted to platelets, nor was it unique to the α_2A_-subtype, as signaling of the three human α_2_-adrenoceptor subtypes (α_2A_, α_2B_, and α_2C_) was potentiated by GSNO in CHO cell lines stably expressing these receptors (our unpublished observations).

### RSNOs amplify muscarinic responses by increasing the rate of GDP/GTP exchange and the number of high-affinity GTP binding sites

The amplification of M2/M4 responses by RSNOs was clearly evident in native brain tissue. Further experiments were designed to address the mechanism of this action. Results of these studies are presented in Figures 6 and 7. A time-response study on basal and CCh-stimulated [^35^S]GTPγS binding responses in forebrain membranes revealed that GSNO accelerated the rate of [^35^S]GTPγS binding in agonist-stimulated conditions (Figure 6). In contrast, GSNO had no effect on basal guanine nucleotide exchange. Given that GDP release is generally thought to be the rate-limiting step in receptor-driven G protein activation, these data indicate that the amplifying effect of GSNO on M2/M4 receptor-stimulated G protein activity is due to an accelerated rate of GDP/GTP exchange at the receptor-activated G protein α subunits. In line with this, GSNO significantly increased the number of high-affinity [^35^S]GTPγS binding sites available for M2/M4 receptor activation under agonist-stimulated conditions (Figure 7) (Mean ± SE: CCh-control 1.49 ± 0.02 vs. CCh-GSNO 1.75 ± 0.02 pmol/mg protein, P < 0.001, unpaired T-test). In contrast, there were no statistical differences in the potency of GTPγS to displace [^35^S]GTPγS in any of the tested conditions (Figure 7) [log (EC_50_) ± SE: basal-control -8.10 ± 0.07; basal-GSNO -8.11 ± 0.12; CCh-control -8.06 ± 0.06; CCh-GSNO -8.10 ± 0.04). These experiments indicate that RSNOs amplify muscarinic receptor-stimulated G protein activity in native brain tissue by accelerating the rate by which agonist-occupied receptors can activate their cognate G proteins.

### RSNO amplification of M4 receptor responses are preserved under heterologous expression but the amplification is lost with constitutive receptor activity

**Figure 8:**
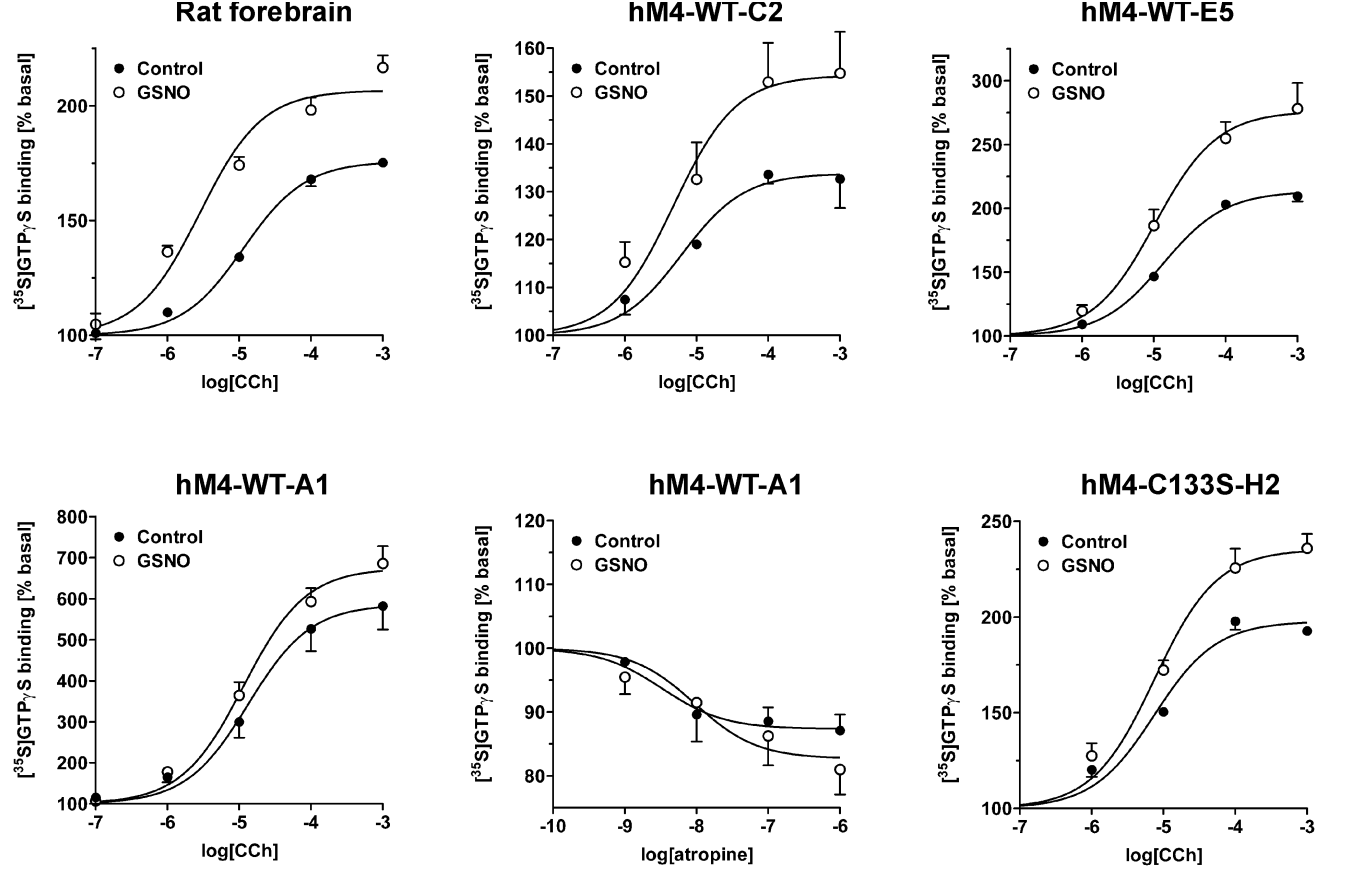
**GSNO-evoked potentiation of muscarinic signaling is preserved under heterologus expression but the effect diminishes with increasing constitutive activity**. The human M4 (hM4) receptor was stably transfected into CHO cells and wild type (WT) or mutant (C133S) cell lines with differential G protein activation capacity were compared with that in rat forebrain membranes. CCh-stimulated G protein activity was determined in control conditions and in membranes pretreated for 30 min with 0.5 mM GSNO, as detailed in the *Methods *section. Note that cell lines WT-C2, WT-E5 and C133S-H2 have maximal responses comparable to that in native brain tissue. Note also robust G protein activation in WT-A1 cell line, as well as its constitutive activity, as evidenced by the ability of the inverse agonist atropine to inhibit basal G protein activity in this cell line. Potency and efficacy values are summarized in Tables 1 (rat brain) and 2 (hM4 clones). Values are mean ± SE from at least three independent experiments performed in duplicate.

To investigate the signaling of the human M4 receptor (hM4) under heterologous expression system, the receptor was stably transfected into CHO cells. The effects of GSNO on agonist-stimulated G protein activity were compared in rat forebrain membranes and three cell lines expressing the wild-type (WT) hM4 receptor with increasing capability to activate G proteins. The results of these experiments are shown in Figure 8, and the potency and efficacy values are summarized in Table 2. In control conditions, CCh stimulated [^35^S]GTPγS binding in the three WT-hM4 cell lines at a similar potency but varying efficacy (E_max _ranging from 134 to 586 %) (Figure 8, Table 2). GSNO treatment significantly increased the E_max _in the two WT-hM4 cell lines (E5 and C2) that showed comparable maximal responses to the values obtained in rat forebrain membranes (Figure 8, Table 2). In contrast, the potentiating effect of GSNO was lost in the WT-hM4-A1 cell line, where CCh robustly activated G proteins (Figure 8, Table 2). In contrast to the situation in brain membranes and other hM4 cell lines of this study, WT-hM4-A1 cell line exhibited constitutive activity, i.e. significant G protein activation was evident in the absence of added agonist. In this cell line, the mAChR antagonist, atropine, significantly inhibited basal G protein activity by 13 ± 3 % (mean ± SE, n = 3, P < 0.05) (Figure 8, bottom panel middle). The effect was dose-dependent [log(IC_50_) -8.48 ± 0.23 (mean ± SE, n = 3)], indicating increased constitutive activation of WT-hM4 receptor in this cell line. Collectively these experiments indicate that the potentiating effect of GSNO on hM4 receptor responses was preserved under heterologous expression but that the effect was diminished with constitutive receptor activity.

The final experiments were intended to clarify whether cysteine 133 (C133), located adjacent to the G protein-interacting DRY-motif in the intracellular end of transmembrane helix 3 of the G_i_-coupled muscarinic receptors (M2/M4), could serve as the molecular target of RSNO action. To this end, C133 was mutated into serine to reveal if the C133S mutation would abolish the effect of GSNO on the efficacy of CCh. However, GSNO treatment significantly increased E_max _also in the mutant C133S-hM4 cell line (Figure 8, bottom panel right, Table 2), indicating that cysteine C133 of the hM4 receptor is not the specific target of RSNOs. The mutation did not affect the potency of CCh to activate G proteins via the hM4 receptor (Table 2).

## Discussion

[^35^S]GTPγS autoradiography of brain cryostat sections revealed a highly receptor-specific modulation of GPCR signaling by RSNOs in several receptor-enriched anatomical structures. This modulation was fully reversible upon addition of excess thiols. We have provided evidence indicating that S-nitrosylation, rather than other types of NO reactions or the NO – guanylyl cyclase – cGMP signaling pathway, was responsible for the observed effects. The RSNOs effects were receptor-specific, as signaling of some receptors was markedly potentiated (M2/M4 AChRs and α_2_-adrenoceptors), whereas that of others was clearly inhibited (P2Y_12_, LPA and cannabinoid CB_1 _receptors), while signaling of other receptors was only marginally affected (adenosine A_1_, MOR, and ORL1 receptors) by comparable treatments. We further demonstrated that RSNOs can amplify M2/M4 receptor responses by increasing the rate of GDP/GTP exchange as well as the number of high-affinity G protein α subunits capable of interacting with the agonist-activated receptors. The potentiating effect of RSNOs on hM4 receptor responses was preserved when this was studied in a heterologous expression system but was diminished in constitutively active hM4 receptors. We also demonstrated that the GPCR itself or its native signaling partners serve as potential targets of this modulation, as it was attenuated, or even lost, when receptor signaling was studied under heterologous expression. Our study suggests that GPCR signaling is subject to a highly receptor-specific modulation by NO-derived RSNOs.

### The GPCRs and their proximal signaling partners as likely targets of RSNO action

Since [^35^S]GTPγS binding assays monitor G protein activation, one of the earliest measurable steps in GPCR signal transduction, it is obvious that the molecular targets of RSNO action are the receptors, their cognate G proteins and/or additional signaling partners, whose thiol modification can directly regulate guanine nucleotide binding and hence G protein activation.

It is interesting that RSNO treatment of brain sections consistently resulted in thiol-sensitive increases in basal G_i _protein activity throughout the gray matter regions. However, no such effect was present in brain membrane [^35^S]GTPγS binding assays, nor was it detected in CHO cell membranes but in platelet membranes, RSNOs inhibited basal G protein activity by ~20%. As receptor input should be minimal in basal conditions, the differential behavior of RSNOs in cryostat sections and various membrane preparations likely reflects direct action on the G_i _proteins and/or their proximal regulatory partners. It has been known for some time that G proteins can serve as direct targets of ROS, including NO [21-24,28,29]. Exogenous NO donors stimulate the monomeric G protein p21^ras ^via S-nitrosylation to a single cysteine residue [22,53]. Furthermore, Gα_i _and Gα_o _serve as direct protein targets of ROS, and they can be activated in the absence of input from the GPCRs [29,33]. Specifically, ROS were shown to modify two cysteine residues of Gα_i/o _and this modification accelerated GDP release from Gα with a concomitant increase in the formation of the GTP-bound form of Gα without receptor activation [29,33]. Furthermore, GSNO was reported to stimulate basal G protein activity in bovine aortic endothelial cells and human peripheral blood mononuclear cells [22,26]. The RSNO-elicited increase in basal G protein activity in brain sections is consistent with these findings. However, it is not clear why no such stimulation was detected in bulk membrane preparations. One explanation could be the differential accessibility of target thiol(s) in cryostat sections as compared to membrane preparations. It is reasonable to assume that thiol accessibility in the former situation is close to natural. It is also possible that some crucial regulatory component of the signaling machinery is lost in bulk membrane preparations. Since several high-speed centrifugation steps were employed to obtain the relatively pure membrane preparation used in our study, this possibility should not be underestimated. This could also explain why RSNOs had no effect on basal G protein activity in CHO cell membranes, although several members of the G_i _family, including Gα_i1/2_, Gα_i3_, and Gα_o_, are endogenously present in these cells [54].

In platelet membranes, RSNOs inhibited both basal and P2Y_12 _receptor-dependent G protein activity, but clearly potentiated α_2A_-adrenoceptor responses. Platelet P2Y_12 _receptors couple to Gα_i2 _[51,52], whereas α_2A_-adrenoceptors can communicate via Gα_z_, at least in the mouse [55]. Collectively these data suggest that RSNOs inhibit both basal and receptor-stimulated Gα_i2 _activity in the platelets. It is not yet known whether brain P2Y_12 _receptors couple to this particular G protein subtype. Further, it is unclear whether LPA receptors in brain and CHO cells communicate via Gα_i2_. It is interesting that RSNOs inhibited the signaling of all three receptors in native tissues. In addition, we found that brain cannabinoid CB_1 _receptor signaling was inhibited by RSNOs. A previous study reported that pulmonary vasoconstriction by serotonin was also inhibited by GSNO [34].

Although the basic module of GPCR signaling is traditionally considered to be the receptor, its cognate G protein, and the effector, recent studies have identified a wide range of proteins that can directly interact with the receptor and/or G proteins. These can modulate signaling efficiency, cellular localization, or the regulation of the GPCRs or G proteins [6]. One such recently-identified protein is the brain-enriched, Ras-related monomeric G protein Dexras1 (human counterpart is termed activator of G protein signaling 1, AGS1). Dexras1/AGS1 is physiologically activated upon NMDA receptor-stimulated NO synthesis and S-nitrosylation on cysteine C11 [56,57]. Dexras1/AGS-1 also interacts with Gα_i_/Gα_o_, and can activate GPCR signaling systems independently of receptor input [6,58]. Interestingly, Dexras1/AGS-1 was shown to proximally antagonize the signaling of M2 AChRs and formyl peptide receptors, possibly by altering the pool of G proteins available for receptor coupling and/or disruption of a preformed signaling complex [59,60]. It is currently unknown whether RSNOs and/or S-nitrosylation could alter the ability of Dexras1/AGS-1 to modify G protein function and/or input from the GPCRs. In light of the present findings, this should be an attractive hypothesis for future studies.

Most Gα proteins are palmitoylated at a cysteine near the amino terminus and this modification is required for G protein targeting to lipid rafts [61] and/or normal signaling [62]. Addition and removal of the palmitoyl group appear to be dynamic receptor-mediated processes that may contribute to recycling of Gα between the membrane and cytosolic compartments [63]. Since our experiments used only nonliving tissue, it is unlikely that the RSNO actions would achieve such extensive lipid modifications, although such alterations reportedly occur after exposure of living cells to NO [64].

### Functional implications

Specialized plasma membrane microdomains act as unique platforms with specific enrichment of GPCRs, their signaling partners, and the enzymatic machinery for NO biosynthesis [35,37-40]. Such close proximity of the GPCR signaling complex and NO source is particularly relevant for many of the receptors whose signaling was shown here to be reversibly modulated by RSNOs.

In the heart, endothelial NOS (eNOS) is localized n caveolin-enriched myocyte membrane fractions and it has been shown that lipid draft-disrupting agents severely compromise NO-dependent inhibition of adenylyl cyclase types 5 and 6 [35]. M2-mediated parasympathetic cardiac signaling also critically involves eNOS activation and NO production [65]. On the other hand, caveolar sequestration of M2 receptors and NO signaling was shown to be finely tuned in the myocytes [66], suggesting a dynamic interplay between the M2 receptor and NO. In hM4 expressing CHO cells, SNP induced agonist-independent internalization of the receptor via atropine- and thiol-sensitive mechanisms [67]. Furthermore, ROS including NO, potentiate cardiac M2 receptor signaling via poorly defined mechanisms [32,68]. Consistent with these findings, our study revealed robust amplification of M2/M4 receptor signaling by RSNOs both in the brain and in CHO cells expressing the hM4 receptor. In CHO cells, however, the RSNO effect clearly diminished with increasing constitutive receptor activity, suggesting that RSNO action and constitutive receptor activity likely share common mechanisms, including accelerated GDP/GTP exchange in cognate Gα subunits. As far as we are aware, this is the first study to show robust, and highly region-specific amplification of M2/M4 receptor signaling in discrete anatomical loci of the central nervous system. The functional consequences of these findings remain to be established.

The P2Y_12 _receptor plays a central role in platelet activation and aggregation [69]. Previous studies have indicated that endothelial and platelet-derived NO, as well as exogenous RSNOs, are potent inhibitors of platelet aggregation [70-73]. Both cGMP-dependent and -independent mechanisms and several potential molecular targets have been implicated in these effects [72,74]. A previous report provided evidence for cGMP-mediated signaling in the inhibition of platelet G_i _signaling [74]. The present study adds further dimensions to this scheme by demonstrating that RSNOs can inhibit platelet (and brain) P2Y_12 _receptor function via cGMP-independent mechanisms, likely involving S-nitrosylation. However, since the effect was lost in CHO cells stably expressing the hP2Y_12 _receptor, the native signaling partners, rather than the P2Y_12 _receptor, serve as obvious targets of this action.

One of the novel findings in this study was that RSNOs strongly inhibited G_i_-mediated LPA receptor signaling in the brain and in CHO cells. The relevance of this finding with respect to brain LPA_1 _receptor signaling remains to be established. In vivo, peripheral LPA receptor signaling is closely associated with NO. In bovine aortic endothelial cells, LPA stimulates endothelial NOS via G_i_-coupled LPA receptors [75]. LPA is released from activated platelets and this stimulates other platelets to activate aggregation processes [76,77]. Inhibition of LPA signaling via endogenous RSNOs could be one important mechanism by which endothelium-derived NO can suppress LPA-mediated athero- and thrombogenic signaling.

## Conclusion

In conclusion, this study revealed that G protein activation, an early step of GPCR signal transduction, is subject to a reversible and highly receptor-specific modulation by exogenous RSNOs at physiologically relevant concentrations. Since NOS synthases (and thus NO production) have been shown to reside in close proximity with the GPCR signaling machinery, especially for many of the receptors whose signaling is subject to modulation by exogenous RSNOs, these findings suggest that GPCR signaling in vivo is likely to be finely tuned by NO-derived RSNO species. Future studies should aim at pinpointing the precise molecular targets of these actions, and at understanding the specific modifications (S-nitrosylation and/or S-thiolation) involved, as well as revealing the physiological and/or pathophysiological relevance in vivo.

## Methods

### Materials

All drugs and chemicals were from Sigma (St. Louis, MO) or Merck (Darmstadt, Germany), unless otherwise stated. Cell culture media, sera, and antibiotics were from Euroclone (Pero, Italy). Protein concentrations were determined with Bio-Rad protein assay (Bio-Rad, Hercules, CA, USA). Adenosine deaminase (ADA) was purchased from Roche (Mannheim, Germany) and guanosine-5'-O-(3-[^35^S]-thio)-triphosphate ([^35^S]GTPγS; initial specific activity 1250 Ci/mmol) from NEN (Boston, MA). CP55940, DAMGO, nociceptin, and SNAP were purchased from Tocris Cookson Ltd. (Bristol, UK).

### DNA constructs

Human M4 muscarinic receptor (hM4, gift from Dr. Johnny Näsman, University of Kuopio) was subcloned from pBluescript into pcDNA3 mammalian expression vector and a triple hemagglutinin (HA) epitope tag was subcloned after the initiating Met codon of the hM4 gene. This construct was used to create a C133S mutant hM4 receptor with QuickChange Site Directed Mutagenesis Kit (Stratagene, La Jolla, CA). Human P2Y_12 _purinergic receptor (hP2Y_12_) was amplified from QuickClone human brain cDNA (Stratagene) using RT-PCR with gene-specific primers. The PCR product was ligated into pcDNA3 and a N-terminal hemagglutinin (HA) epitope tag was inserted in a PCR reaction with 5' primer containing the HA tag DNA sequence. All receptor constructs were confirmed by restriction analyses and DNA sequencing prior to transfections.

### Cell culture and transfection

Recombinant plasmids were introduced into Chinese hamster ovary (CHO) cells with Lipofectamine 2000 transfection reagent (Gibco, Paisley, UK). Transfected cells were placed under G-418 selection (600 μg/ml) and several cell lines originating from single G-418 resistant cells were isolated. The G-418 resistant cell lines were cultured as monolayers with 100 μg/ml G-418 in Ham's F-12 nutrient mixture, containing 10% fetal calf serum, 100 U/ml penicillin and 100 μg/ml streptomycin at 37°C in a humidified atmosphere of 5% CO_2 _/ 95% air. Stable cell lines were analyzed for HA tag (and thus receptor) expression using receptor ELISA with mouse anti-HA primary antibody [78]. The cell lines that showed high receptor expression levels in receptor ELISA were maintained for subsequent experiments. From these, several hM4 and hP2Y_12 _cell lines were established that responded to carbachol (CCh) or 2-methylthio-ADP (2MeSADP), respectively. Nontransfected CHO cells did not respond to CCh (data not shown). CHO cells endogenously express G_q_-coupled P2Y receptors (P2Y_1 _and P2Y_2_). However, CHO cells that had not been transfected with the hP2Y_12 _receptor construct did not respond to 2MeSADP, indicating that activation of G_q _was not detected using [^35^S]GTPγS binding assays (data not shown).

### Preparation of cryostat sections, membranes, and cell lysates

Naïve, four-week-old male Wistar rats were used for the preparation of brain cryostat sections essentially as described earlier [41,79]. All animal protocols were approved by the local ethics committee. Platelet membranes were prepared from expired human platelets (Red Cross, Helsinki, Finland) as previously described [42], except that protease inhibitor cocktail was omitted. CHO cells were harvested by scraping in PBS containing 5 mM EDTA. Membrane fractions (P2) from rat brains and hM4 cell lines were isolated with differential centrifugation using previously published protocols [80,81]. For membrane preparation from hP2Y_12_-expressing cell lines, the centrifugation method used for platelet membrane preparation [42] was used, with the modification of omitting the protease inhibitor cocktail. For the preparation of whole-cell lysates, the cells were harvested by trypsinization in normal growth medium. Cell density was counted in a hemocytometer and the cells were pelleted by centrifugation for 10 min at 250 × g at room temperature. The cell pellets were washed with PBS and centrifuged twice as above, after which the dry pellets were snap-frozen on dry ice. The frozen pellets were thawed for 1 min in a water bath at room temperature, after which snap-freezing was repeated. The final membrane and lysate preparations (1–5 mg protein/ml) were stored as single-use aliquots in -75°C.

### Preparation of S-nitrosothiols

S-nitroso-N-acetyl-D,L-penicillamine (SNAP) was purchased from Tocris Cookson Ltd. (Bristol, UK). All other RSNOs were synthesized from the respective thiols using acidified NaNO_2_. For example, S-nitrosoglutathione (GSNO) was prepared by mixing 100 μl sodium nitrite (100 mM) with 100 μl HCl (150 mM) and adding 100 μl reduced glutathione (100 mM). Reactions were allowed to proceed for 10 min at room temperature, protected from light. Reaction mixtures were neutralized with 150 μl NaOH (100 mM) and used immediately in the experiments. Millipore-quality water was used throughout and the assay buffer routinely contained 1 mM EDTA. The concentrations of RSNOs were determined by UV spectroscopy using previously published [70] values for the molar absorption coefficients (ε) and absorption maxima (λ_max_).

### [^35^S]GTPγS autoradiography

The assay was conducted under optimized conditions, where basal noise due to tonic adenosine A_1 _receptor activity has been eliminated [79]. Experiments were conducted in light-protected chambers and in the absence of dithiotreitol (DTT), unless indicated otherwise. Briefly, the assay consisted of preincubation for 20 min at 20°C in buffer A (50 mM Tris-HCl, pH 7.4, 1 mM EDTA, 100 mM NaCl, 5 mM MgCl_2_), followed by GDP loading and RSNO treatment for 1 h at 20°C in buffer A, containing additionally 2 mM GDP and 8-cyclopentyl-1,3-dipropylxanthine (DPCPX, 10^-6 ^M) or adenosine deaminase (ADA, 1 U/ml) to eliminate tonic adenosine A_1 _receptor activity. When peptide agonists were used, protease inhibitor cocktail (Sigma P-2714) was included in step 2 at the concentrations recommended by the manufacturer. For [^35^S]GTPγS binding, sections were incubated for 90 min at 20°C in buffer A, containing additionally 80 pM [^35^S]GTPγS, 2 mM GDP, DPCPX (10^-6 ^M) or ADA (1 U/ml), and the receptor agonists and/or reduced thiols (DTT, GSH or cysteine), as detailed in the results section. Nonspecific binding (Nsb) was determined in the presence of 10 μM GTPγS. The sections were washed twice at 0°C for 5 min in washing buffer (50 mM Tris-HCl, 5 mM MgCl_2_, pH 7.4), rinsed in ice-cold deionized water for 30 s, air dried and apposed to Biomax™ MR film (Kodak) for 6–11 days. Autoradiography images were digitized and processed for figures, as previously described [41].

### [^35^S]GTPγS membrane binding assays

The incubations were carried with slight modifications to previously published protocols [80,81]. Membranes or lysates were preincubated for 30 min at room temperature in 50 mM Tris-HCl (pH 7.4), 1 mM EDTA, 100 mM NaCl, 5 mM MgCl_2_, 10 μM GDP and 0.5 U/ml ADA, under constant shaking and protected from light. RSNOs (usually GSNO or CysNO) were included in the preincubation at final concentrations of 0.5 mM. The assay was performed in duplicate in a final assay volume of 400 μl. The reaction was initiated by adding 40 μl of membrane or lysate preparation (5 μg protein or 50,000 cells / tube) to incubation tubes containing drug dilutions and binding cocktail. The final concentrations of the components in binding reaction were 50 mM Tris-HCl (pH 7.4), 1 mM EDTA, 100 mM NaCl, 5 mM MgCl_2_, 0.5% BSA, 10 μM GDP, 0.5 U/ml ADA and 150 pM [^35^S]GTPγS. Nsb was defined using 10 μM GTPγS. Reaction tubes were incubated for 90 min at 25°C under constant shaking. The reaction was quickly terminated by the addition of 4 ml ice-cold wash buffer (50 mM Tris-HCl (pH 7.4), 5 mM MgCl_2_) followed by rapid filtration through Whatman GF/B glass fiber filters (Whatman, Maidstone, UK) and two additional 4 ml washes with the buffer. In the time-response study (Figure 6), initial reaction volume was 2 ml and aliquots (400 μl) from duplicate samples were draw at different time points (15, 30, 60 and 90 min), and the reaction was terminated as described above. Radioactivity in filters was counted with Wallac Rackbeta liquid scintillation counter (Wallac, Turku, Finland). It should be noted that RSNO treatment caused a small, yet consistent increase in Nsb (25 ± 4 %, mean ± SEM, n = 13). However, as Nsb represented <0.3 % of total radioactivity, this effect was considered negligible and did not contribute to the results and conclusions thereof.

### Data analysis

[^35^S]GTPγS-membrane binding data were analyzed with GraphPad Prism software (GraphPad, San Diego, CA) using non-linear fitting for sigmoid dose-response curves. Statistical analyses were made with one-way analysis of variance (ANOVA) followed by Tukey's multiple comparison test. When comparison was made between only two groups, unpaired T-test was used.

## List of abbreviations

2MeSADP, 2-methylthio-ADP; [^35^S]GTPγS, guanosine-5'-O-(3-[^35^S]-thio)-triphosphate; 5-HT, 5-hydroxytryptamine; ADA, adenosine deaminase; AGS, activator of G protein signaling; CCh, carbacholine; CHO, Chinese hamster ovary; CP-55940, (-)-3-[2-hydroxy-4-(1,1-dimethylheptyl)-phenyl]-4-[3-hydroxypropyl]cyclohexan-1-ol; CysNO, S-nitrosocysteine; CysNO-Gly, S-nitroso-cysteinyl-glycine; DAMGO, [D-Ala^2^, N-Me-Phe^4^, Gly^5^-ol]-enkephalin; DPCPX, 8-cyclopentyl-1,3-dipropylxanthine; DTT, dithiotreitol; Glu-CysNO, L-γ-glutamyl-S-nitrosocysteine; GSH, glutathione; GPCR(s), G protein-coupled receptor(s); GSNO, S-nitrosoglutathione; HA, hemagglutinin; hM4, human muscarinic receptor subtype 4; hP2Y_12_, human P2Y_12 _purinergic receptor; LPA, lysophosphatidic acid; NA, noradrenaline; NO, nitric oxide; NOBF_4_, nitrosodium tetrafluoroborate; NOS, NO synthase; RGS, regulator of G protein signaling; RT, reverse transcriptase; SNAP, S-nitroso-N-acetyl-D,L-penicillamine; RSNO, S-nitrosothiol; SNP, sodium nitroprusside

## Authors' contributions

TK carried out the cell culture, molecular biology and mutagenesis studies, participated in the membrane and lysate [^35^S]GTPγS binding assays, participated in the design of the study and drafted the manuscript. JRS and MDR carried out most of the membrane [^35^S]GTPγS binding assays and performed the statistical analyses for these. KSM carried out G protein activation assays with human platelets. JTL conceived of the study, its design and coordination and conducted [^35^S]GTPγS autoradiography experiments. All authors read and approved the final manuscript.

## Supplementary Material

Additional File 1 Supplementary material (Supplementary Figures 1, 2, 3, 4, 5, 6, 7 and Supplementary Tables 1, 2) is provided as a single file. This pdf-file (size 0.75 MB) is readable using Adobe Acrobat. Click here for file
