# Supplementary material for: S-Nitrosothiols modulate G protein-coupled receptor signaling in a reversible and highly receptor-specific manner
Source: BMC Cell Biol. 2005 Apr 25;6:21. doi: 10.1186/1471-2121-6-21 (PMC1090567; doi:10.1186/1471-2121-6-21)
Supplement: Additional File 1 — Supplementary material (Supplementary Figures 1, 2, 3, 4, 5, 6, 7 and Supplementary Tables 1, 2) is provided as a single file. This pdf-file (size 0.75 MB) is readable using Adobe Acrobat. [file 1471-2121-6-21-S1.pdf]

Supplementary material

accompanying the manuscript *"S-Nitrosothiols modulate G protein-coupled receptor signaling in a reversible and highly receptor-specific manner"*

by

Tarja Kokkola, Juha R. Savinainen, Kati S. Mönkkönen, Montse Durán Retamal, and Jarmo T. Laitinen

---

Content:

Supplementary Figures 1-7 (pages 2-8)

Supplementary Tables 1-2 (pages 9-10)

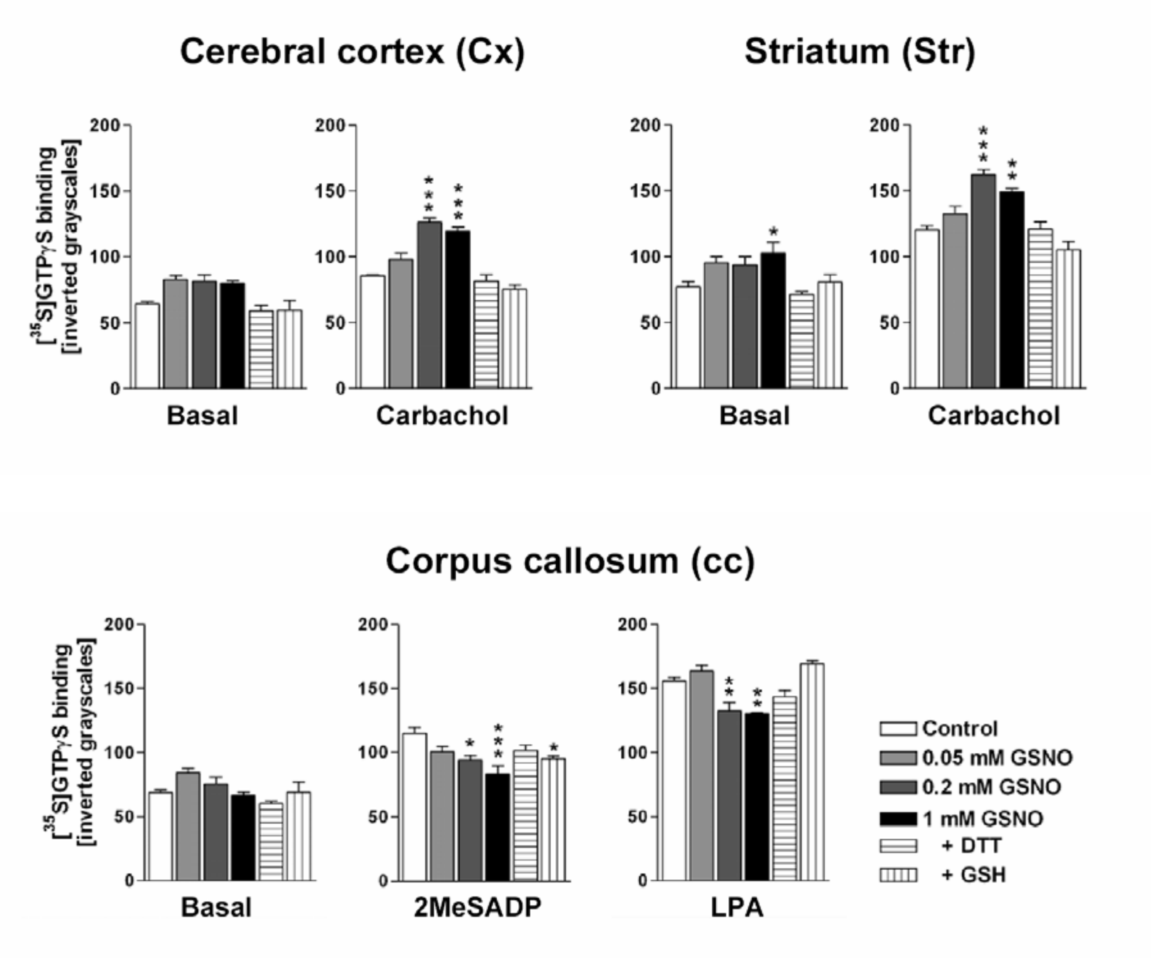

Supplementary Figure 1.

Quantitative data on the dose-dependent and reversible effects of GSNO on basal and receptor-dependent [^{35}S]GTPγS binding responses in selected brain regions. [^{35}S]GTPγS autoradiography was conducted, as detailed in the *Methods* section. GSNO (0.05-1 mM), DTT (1 mM) or GSH (1 mM) were present, as indicated. Agonist stimulated responses were evaluated using carbachol (10^{-4} M), 2MeSADP (10^{-6} M) or LPA ($5x10^{-5}$ M in 0.1 % fatty acid free BSA). Autoradiography images representing coronal sections (approximate Bregma level -0.92 - -2.8 mm) were digitized and regional grayscale values were obtained for selected regions from the inverted images after subtraction of nonspecific binding. In this presentation, increase in [^{35}S]GTPγS binding results in higher grayscale values. Values are mean + SE representing coronal sections from four individual animals. Statistical comparisons were made using one way analysis of variance (ANOVA) followed by Tukey's multiple comparison test. Risk levels: * P<0.05; ** P<0.01; *** P<0.001.

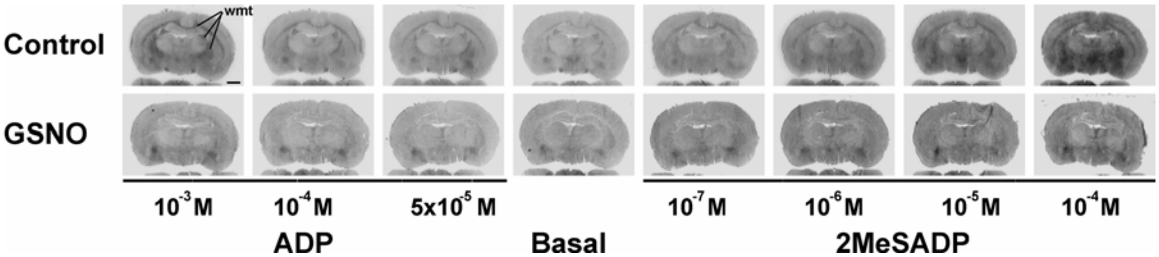

Brain regional [^{35}S]GTPγS binding responses [% basal, mean ± SE, n=4]

	Corpus callosum		Cerebral cortex		Hippocampus *Stratum lacunosum-moleculare*		Overall coronal plane	
	Control	GSNO	Control	GSNO	Control	GSNO	Control	GSNO
**Basal**	100 ± 5	100 ± 2	100 ± 5	100 ± 5	100 ± 4	100 ± 2	100 ± 2	100 ± 4
	(55 ± 3	53 ± 1	54 ± 3	72 ± 3**	53 ± 2	76 ± 1***	56 ± 1	68 ± 3**)
**ADP**								
$5x10^{-5}$ M	107 ± 4	87 ± 7*	95 ± 4	84 ± 6	98 ± 2	83 ± 5*	114 ± 3	82 ± 2***
10^{-4} M	127 ± 3	102 ± 4**	109 ± 3	98 ±12	111 ± 4	97 ± 6	125 ± 1	95 ± 5**
10^{-3} M	194 ±11	134 ± 8**	145 ±14	107 ± 6*	164 ± 9	92 ± 8***	153 ± 3	99 ± 3***
**2MeSADP**								
10^{-7} M	131 ± 8	122 ± 7	113 ± 5	108 ± 3	128 ± 7	103 ± 4*	129 ± 4	114 ± 2*
10^{-6} M	154 ± 6	131 ± 5*	129 ± 9	111 ± 4	138 ± 5	107 ± 5**	155 ± 3	118 ± 3***
10^{-5} M	176 ± 4	141 ± 9*	160 ± 9	125 ± 5*	162 ± 9	117 ± 7**	167 ± 5	132 ± 4**
10^{-4} M	212 ± 9	148 ± 6**	184 ± 7	130 ± 4***	200 ± 9	116 ± 3***	208 ± 4	132 ± 6***

* Statistically different from control ($P<0.05$)
** Statistically different from control ($P<0.01$)
*** Statistically different from control ($P<0.001$)

Supplementary Figure 2.

GSNO inhibits $P2Y_{12}$ receptor responses in the rat brain throughout all receptor-responsive regions. Top: [^{35}S]GTPγS autoradiography was conducted, as detailed in the *Methods* section. Where indicated, GSNO (1 mM) was present. The cognate $P2Y_{12}$ receptor agonists ADP or its potent analog 2MeSADP were present at the indicated concentrations. Note that GSNO blunts $P2Y_{12}$ receptor responses for both agonists, and in all brain regions. This effect is most prominent in the white matter tracts (wmt). Scale bar = 2 mm. Bottom: Quantitative data of [^{35}S]GTPγS binding responses in selected brain regions. Autoradiography images representing coronal sections (approximate Bregma level -3.8 - -5.2 mm) were digitized and regional grayscale values were obtained from the inverted images. In this presentation, increase in [^{35}S]GTPγS binding results in higher grayscale values. Values are mean ± SE and derived from coronal sections of four individual animals. Statistical comparisons (unpaired T-test) were made between control and GSNO treatments for each agonist concentration. Grayscale values for the basal condition are shown in parenthesis.

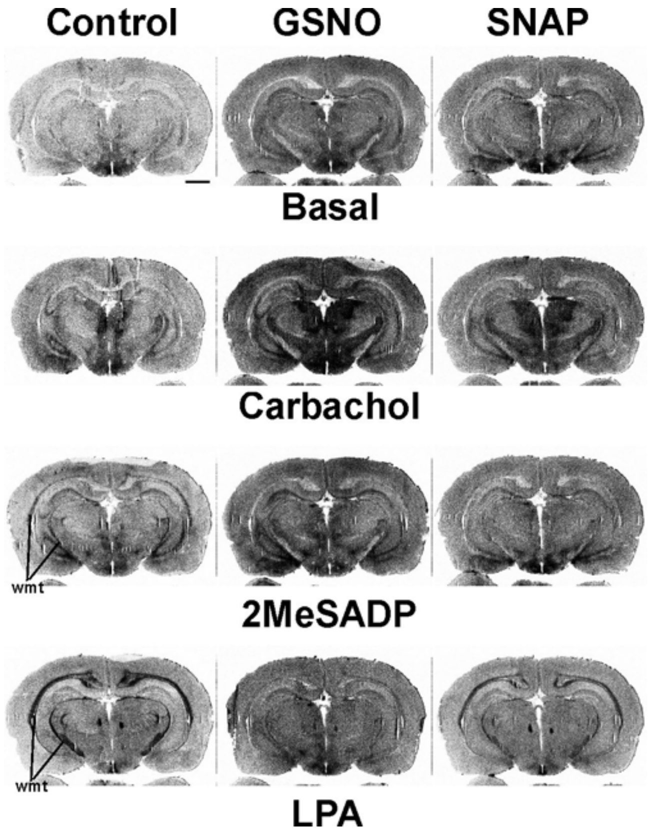

Supplementary Figure 3.

S-nitroso-N-acetyl-D,L-penicillamine (SNAP) mimics S-nitrosoglutathione (GSNO) in modulating basal and receptor-dependent G protein activity in rat brain cryostat sections. [^{35}S]GTPγS autoradiography was conducted, as detailed in the *Methods* section. Where indicated, GSNO or SNAP (0.5 mM each) were present. The GPCR agonists carbachol (10^{-4} M), 2MeSADP (10^{-5} M), or LPA ($5x10^{-5}$ M in 0.1 % fatty acid free BSA) were used. Note that GSNO and SNAP equally well inhibit $P2Y_{12}$ receptor signaling, an effect that is most evident in the white matter tracts (wmt). Note also that GSNO is more potent than SNAP in amplifying M2/M4 receptor signaling throughout the gray matter regions, and in suppressing LPA-stimulated responses throughout the white matter tracts. Scale bar = 2 mm.

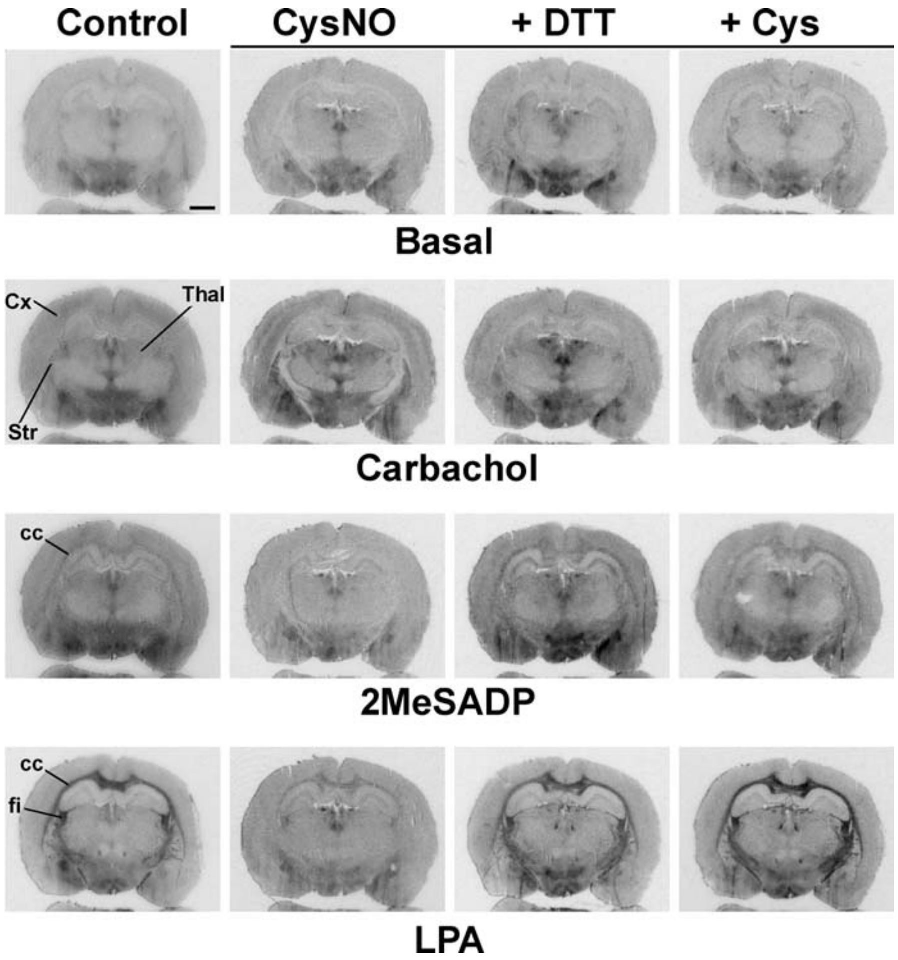

Supplementary Figure 4.

S-nitrosocysteine (CysNO) reversibly modulates basal and receptor-dependent G protein activity in rat brain cryostat sections. [^{35}S]GTPγS autoradiography of coronal brain sections was conducted, as detailed in the *Methods* section. Where indicated, CysNO (1 mM), DTT (1 mM) or cysteine (Cys) (1 mM) were present. The GPCR agonists carbachol (10^{-4} M), 2MeSADP (10^{-6} M) and LPA (5×10^{-5} M in 0.1 % fatty acid free BSA) were used. In the control panel (left), the anatomical loci where receptor agonists typically activate G proteins are indicated. Note CysNO-dependent robust amplification of CCh-stimulated G protein activity in several gray matter regions visible at this coronal plane, most notably the striatum (Str), various nuclei of the thalamus (Thal), and the cerebral cortex (Cx). Note also clear attenuation of 2MeSADP-stimulated responses, and blunting of LPA-stimulated responses in the white matter tracts, including the corpus callosum (cc) and the fimbria of the hippocampus (fi). Scale bar = 2 mm.

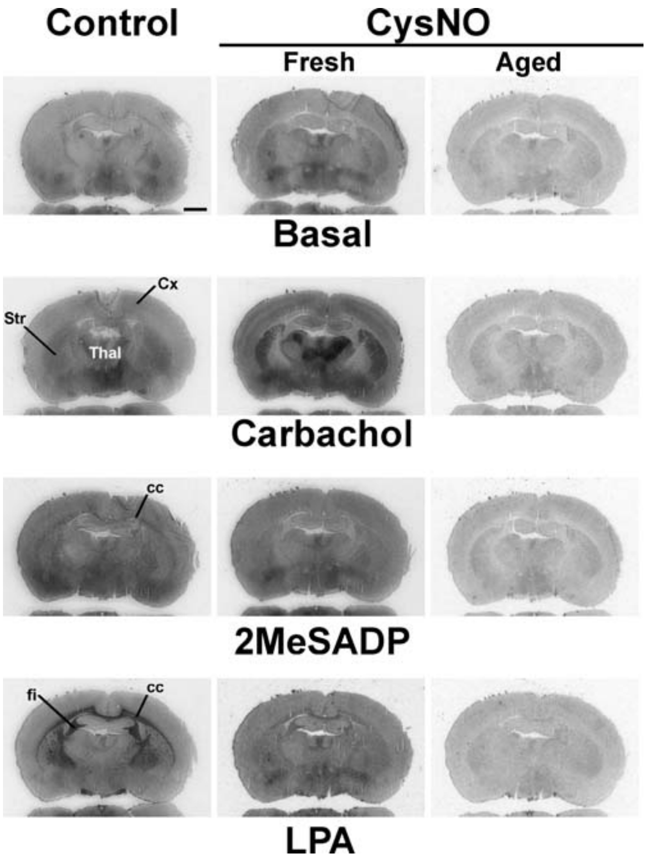

Supplementary Figure 5.

Freshly prepared S-nitroscysteine (CysNO) mimics GSNO in modulating basal and receptor-dependent G protein activity in rat brain cryostat sections whereas aged CysNO prevents receptor-mediated G protein activation. [^{35}S]GTPγS autoradiography was conducted, as detailed in the *Methods* section. Where indicated, freshly prepared or aged CysNO (1 mM each) was present for 60 min during the GDP loading (step 2). Aged CysNO was "prepared" by keeping the stock solution for 2 days exposed to ambient light, oxygen and temperature. The GPCR agonists CCh (10^{-4} M), 2MeSADP (10^{-6} M), or LPA ($5x10^{-5}$ M in 0.1 % fatty acid free BSA) were present in step 3. Note robust amplification of M2/M4 receptor signaling by freshly prepared CysNO, most notably in the striatum (Str), the cerebral cortex (Cx) and various nuclei of the thalamus (Thal). Note also inhibition of $P2Y_{12}$ and LPA_1 receptor signaling by fresh CysNO, an effect that is most evident in the corpus callosum (cc) and the fimbria of the hippocampus (fi). Scale bar = 2 mm.

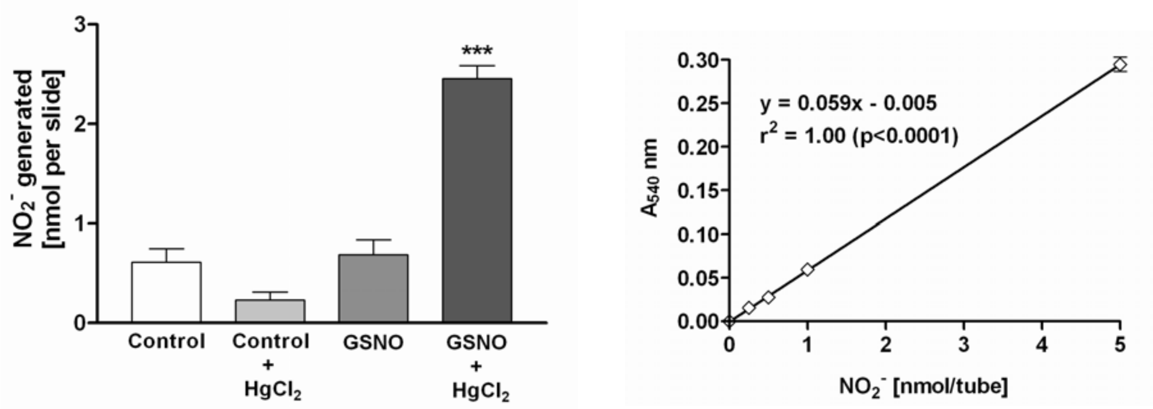

Supplementary Figure 6.

$HgCl_2$-catalyzed cleavage of S-NO bond and subsequent liberation of nitrite from GSNO-treated rat brain sections. Coronal sections (four per slide) were incubated for 20 min at 20 ˚C in TEMN buffer (50 mM Tris-HCl, pH 7.4; 1 mM EDTA; 5 mM $MgCl_2$; 100 mM NaCl; 0.95 ml per slide) and incubated thereafter for 40 min at 20 ˚C in TEMN buffer in the absence (control) or presence of GSNO (0.5 mM). After washing with 50 mM Tris-HCl, pH 7.4 (2 x 0.95 ml per slide), sections were incubated for 80 min in 50 mM Tris-HCl, pH 7.4 in the absence or presence of $HgCl_2$ (10^{-4} M). The supernatant was collected and nitrite determined from duplicate aliquots (0.4 ml) using a colorimetric assay based on the Griess reaction [1]. A protocol with delayed addition of Griess reagent A (naphthylethylenediamine dihydrochloride) was used to enhance assay sensitivity (in our hands <0.25 nmol NO_2^- per tube). The standard line derived from three independent assays is shown in the right panel. Values are mean + SE from triplicate slides and are derived from one of two independent experiments. *** Statistically different from control (P<0.001, one way analysis of variance (ANOVA) followed by Tukey's multiple comparison test).

Reference

1 Cook JA, Kim SY, Teague D, Krishna MC, Pacelli R, Mitchell JB, Vodovotz Y, Nims RW, Christodoulou D, Miles AM, Grisham MB, Wink DA: **Convenient colorimetric and fluorometric assays for S-nitrosothiols** *Anal Biochem* 1996, 238: 150-158

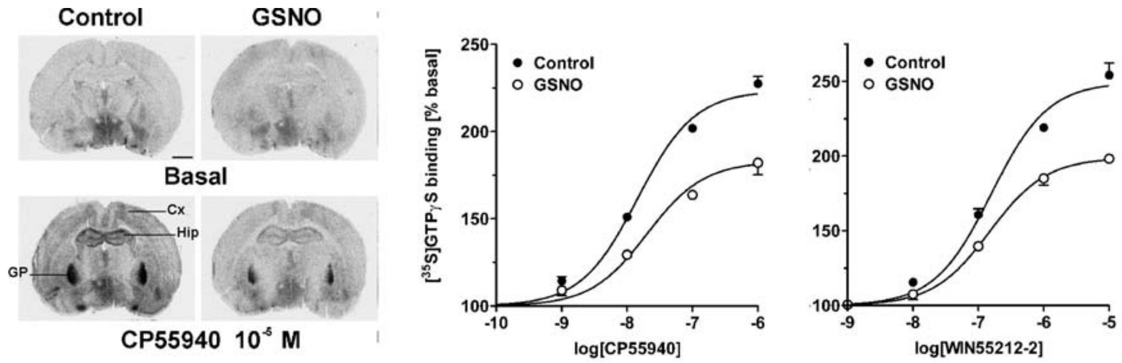

Supplementary Figure 7.

GSNO pretreatment inhibits cannabinoid CB_1 receptor signaling in rat brain cryostat sections and forebrain membrane preparations. [^{35}S]GTPγS binding assays were conducted, as detailed in the *Experimental procedures* section. Note inhibition of responses to the cannabinoid receptor agonists CP55940 throughout all visible CB_1 receptor-enriched regions, including the cerebral cortex (Cx), the globus pallidus (GP), and the hippocampus (Hip). Scale bar = 2 mm.

Supplementary TABLE 1

Brain regional [^{35}S]GTPγS binding responses [% basal, mean ± SE, n=4]

	Control	CysNO	SNP	8Br-cGMP
**Carbachol 10^{-4} M**				
Overall coronal plane	116 ± 4	128 ± 2*	109 ± 3	114 ± 5
Cerebral cortex (Cx)	114 ± 3	134 ± 2*	114 ± 2	115 ± 2
Hippocampus	111 ± 3	123 ± 2*	114 ± 3	113 ± 3
Striatum (Str)	120 ± 5	143 ± 4*	108 ± 3	131 ± 3
Thalamus (Thal)	125 ± 8	137 ± 5	114 ± 8	120 ± 9
**2MeSADP 10^{-6} M**				
Overall coronal plane	128 ± 1	110 ± 1***	115 ± 6	125 ± 3
Corpus callosum (cc)	139 ± 3	123 ± 4*	127 ± 8	134 ± 3
Fimbria of the hippocampus (fi)	118 ± 2	109 ± 1*	106 ± 5	116 ± 5
Cerebral cortex (Cx)	135 ± 6	116 ± 2*	120 ± 4	122 ± 1
Hippocampus	135 ± 3	110 ± 1***	123 ± 6	125 ± 3
Striatum (Str)	127 ± 2	107 ± 1***	103 ± 6**	131 ± 1
Thalamus (Thal)	127 ± 3	107 ± 3**	116 ± 7	126 ± 6
**LPA 5×10^{-5} M**				
Overall coronal plane	126 ± 2	109 ± 1*	118 ± 4	128 ± 1
Corpus callosum (cc)	177 ± 2	143 ± 2***	186 ± 4	169 ± 7
Fimbria of the hippocampus	165 ± 3	152 ± 4*	168 ± 6	166 ± 6

* Statistically different from control ($P<0.05$)
** Statistically different from control ($P<0.01$)
*** Statistically different from control ($P<0.001$)

[^{35}S]GTPγS autoradiography was conducted using a 3-step protocol, as detailed in the *Methods* section. Where indicated, CysNO (1 mM), SNP (0.5 mM), or 8Br-cGMP (0.25 mM) were present for 60 min during the GDP loading step. Autoradiography images representing coronal sections (approximate Bregma level -3.8 - -5.2 mm) were digitized and grayscale values were obtained from the inverted images for selected brain regions. In this presentation, increase in [^{35}S]GTPγS binding results in higher grayscale values. Values are mean ± SE and derived from coronal sections of four individual animals. Statistical comparisons (unpaired T-test) were against the respective control.

Supplementary TABLE 2

Brain regional [^{35}S]GTPγS binding responses [% basal, mean ± SE, n=4]

	Basal		Nociceptin 10^{-6} M		DAMGO 10^{-6} M		2ClAdo 10^{-5} M	
	Control	GSNO	Control	GSNO	Control	GSNO	Control	GSNO
**Cortex**	100 ± 2<br>(63 ± 2	100 ± 11<br>77 ± 8)	172 ± 4	184 ± 4	131 ± 9	136 ± 8	189 ± 4	142 ± 5***
**Striatum**	100 ± 2<br>(78 ± 1	100 ± 4<br>93 ± 4*)	115 ± 2	120 ± 4	128 ± 5	135 ± 4	168 ± 6	132 ± 2**
**Thalamus**	100 ± 4<br>(68 ± 3	100 ± 7<br>86 ± 6*)	161 ±18	152 ±12	149 ±14	152 ±12	254 ± 6	166 ± 5***
**Hippocampus**	100 ± 4<br>(56 ± 2	100 ± 4<br>70 ± 3**)	149 ± 5	150 ± 2	132 ± 4	140 ± 9	274 ± 8	188 ± 3***

* Statistically different from control ($P<0.05$)
** Statistically different from control ($P<0.01$)
*** Statistically different from control ($P<0.001$)

[^{35}S]GTPγS autoradiography was conducted using a 3-step protocol, as detailed in the *Methods* section. Where indicated, GSNO (1 mM) was present for 60 min during the GDP loading step. Autoradiography images representing coronal (approximate Bregma level -2.8 mm) and sagittal sections (approximate lateral plane 3 mm) were digitized and grayscale values were obtained from the inverted images for selected brain regions. In this presentation, enhanced [^{35}S]GTPγS binding is reflected as higher grayscale values. Values are mean ± SE of four individual animals and are derived from two coronal and two sagittal sections. Statistical comparisons (unpaired T-test) were against the respective control. Grayscale values for the basal condition are shown in parenthesis.
